# The non-edible and disposable parts of oyster mushroom, as novel adsorbent for quantitative removal of atrazine and its degradation products from synthetic wastewater

**DOI:** 10.1016/j.heliyon.2024.e26278

**Published:** 2024-02-10

**Authors:** Endale Teju, Abi Legesse, Negussie Megersa

**Affiliations:** a Department of Chemistry, College of Natural and Computational Sciences, Addis Ababa University, P. O. Box 1176, Addis Ababa, Ethiopia; b Department of Chemistry, College of Natural and Computational Sciences, Haramaya University, P. O. Box 138, Haromaya, Ethiopia

**Keywords:** Atrazine, Percent adsorption, Water bodies, Pesticide degradation products, Oyster mushroom, HPLC

## Abstract

In this study, the non-edible part of oyster mushroom was utilized for quantitative removal of the most commonly used *s*-triazine herbicide; atrazine and its breakdown products including deethylatrazine (DEA), hydroxyatrazine (ATOH) and deisopropylatrazine (DIA) from aqueous samples. The functional groups available on the oyster mushroom were studied applying FTIR before and after adsorption. Experimental parameters influencing the uptake process including acidity, sorbent mass, sorption time, initial analyte quantities, and agitation speed were analysed and the maximum removal was found at 4, 0.3 g, 120 min, 0.5 mg L^−1^, and 150 rpm, respectively. Accordingly, the adsorption capacities of 0.994, 1.113, 0.991 and 1.016 mg g^−1^ were obtained for DIA, DEA, ATOH and atrazine, respectively. The adsorption characteristics were discussed utilizing Langmuir and Freundlich isotherm models. The fundamental characteristic of the Langmuir isotherm, which can be elaborated using separation factor or equilibrium parameter, R_L_, and coefficient of variation, R^2^, were (0.761, 0.996), (0.884, 0.975), (0.908, 0.983) and (0.799, 0.984) for DIA, DEA, ATOH and Atrazine, respectively. These findings showed that all analytes' adsorption processes were fitted well to the Langmuir adsorption isotherm, indicating that the adsorbent surface was covered in a monolayer. The kinetics was also evaluated using the pseudo-first and pseudo-second order models. The coefficient of determination, r^2^, were found to be 0.09703, 0.9989, 0.9967 and 0.9998 for DIA DEA, ATOH and atrazine, respectively, for pseudo-second order, signifying that, all analytes were found to follow the pseudo-second order rate model showing that the rate limiting step is chemisorption in the sorption process. Based on these findings, the non-edible and disposable part of the oyster mushrooms can be utilized as a preferred alternative biosorbent for the uptake of the target compounds analysed and other pollutants possessing comparable physicochemical characteristics occurring in various water bodies.

## Highlights

• Disposable mushroom stalk has been introduced, as alternative biosorbent for amenable removal of pesticide pollutants.
• The attractive features of the biosorbent is its easy preparation and modification, and environmentally friendliness.
• Use of this disposable/decomposable adsorbent has the advantage of minimizing chemical pollution loads on environment.
• The experiment requiredcommon laboratory apparatus for achieving quantitative removal of pollutants.
• The available active sites on the stalk surfaces facilitates efficient adsorption of polluting contaminants.

## 1 Introduction

Pesticides are groups of artificially synthesized chemical compounds utilized to control fungi, mites, insects, rodents and weeds attacking the plants, livestock and household animals with the intention of enhancing agricultural production [1]. They are divided into three main classes: herbicides, fungicides, and insecticides. There are also smaller divisions that include acaricides, molluscicides, rodenticides, and nematodes [2]. Following World War II, the application of pesticides in agricultural practices was highly increased with the aim of boosting global food production. Since then, a noticeable evolution has occurred in the variety of pesticide kinds that fall into distinct categories. Despite the remarkable advantages gained, the application of pesticides is also known to cause water quality deterioration in agricultural sector since trace residues and degradation products of these compounds can contaminate the surface as well as groundwaters [3,4].

Application of pesticides is still a major component of agricultural practices worldwide; without it, food production would be severely impacted in terms of both quantity and quality [5]. Furthermore, continuous use of pesticides may also cause undesirable effects on the ecosystem and as a result pesticide residues are detected in various environmental matrices [6]. Despite being prohibited for use in agriculture, different types of pesticides are still being investigated due to their long environmental persistence [7]. Their residues may remain in the soil and may leach into the ground and run-off water bodies [8]. The structure, various moieties linked to the pesticide compounds, the surface configurations, type of the attached molecules, polarity, symmetry and asymmetry of molecules, solubility and sorption properties are the main factors determining a pesticide's toxicity to the pests [9].

These compounds and their metabolites are known to cause serious environmental and human health issues due to their wide range of applications, extensive uses, and physicochemical and toxicological characteristics [4,10]. As a result, concerns regarding environmental protection have increased over the past years all over the world. So far, the penetration of pesticide wastes into the aquifer systems and groundwater tables persists as an unavoidable challenge to public health and food chain interference [11].

In addition to their potential toxicity, persistence, and water solubility, pesticides are utilized extensively, which raises concerns about their analysis in natural waters [12]. They have been determined from a number of environmental matrices such as water [4,8,13], fruit [14], honey [15], fish [16], crops [17], leafy vegetables [18,19], tobacco [20,21], coconut [22], fruit-based soft drinks [23,24], soil [25,26], and ambient air [27,28]. The determinations of pesticides and their metabolites in the aforementioned sample types are indications of the seriousness of the problem in the environment.

Among the most commonly detected pesticides in the water systems, symmetrical *(s*)-triazine herbicides have been known to create environmental risks due to its wide spread use along with their extensive applications, spills and incorrect waste disposal [29,30]. Atrazine; (2-chloro-4-ethylamino-6-isopropylamino-1,3,5-triazine) is a category of *s-*triazine and commonly used all over the world in modern agriculture, due to its broad-spectrum herbicidal properties and low costs to mitigate different types of weeds, mainly in the production of corn and broad leaf plants [17]. Natural and environmental factors including rainfall and temperature known to affect the degradation rate of atrazine in certain soils, making it essential to measure residual levels prior to plant rotational crops [31]. The commonly known atrazine degradation products such as hydroxyatrazine (ATOH), deethylatrazine (DEA) and deisopropylatrazine (DIA) are routinely detected and determined in various environmental compartments and their frequent and regular monitoring is very important, as their toxic effects could also be more serious than the parent compound; atrazine [31].

Following the determination of both the parent compounds and their metabolites, in various sample matrices various analytical methods have been developed and utilized for their efficient removal from streams of environmental wastewaters. The most commonly employed methods include adsorption [8], filtration [16] and membrane process [13]. However, most of these methods have got their own merits and limitations. Because of its simple design for sludge free environment and ability to involve low investment regarding initial cost and requirement of space, adsorption techniques are preferable over other methods [32,33]. Sorption of pesticides onto activated carbon has attracted the attention of several workers [34,35] mainly because of its effectiveness for removal of various organic pollutants at trace levels; however, the high cost of the process restricts its application at larger scales [36,37].

In order to surmount these difficulties, investigation for cheap, environmentally friendly and easily available adsorbent materials for the treatment of contaminants in general, and pesticides in particular, from different water bodies has currently gained prior attention and attracted the interest of researchers. In this study, attempt was made to use the disposable part of mushroom, from a fungus with significant nutritional value, as an adsorbent. Among the various types of edible mushrooms cultivated in Ethiopia, oyster mushroom (*Pleurotus ostreatus)* is the most common one. During its production, lots of waste is generated which demands proper handling to safeguard the environment. The wastes from the mushroom have been utilized in the applications of energy conversion and agriculture, primarily as bioenergy, biocompost and plant growing media [38]. However, there is no report in the literature on its use as toxic pollutant adsorptive removal from water samples. Therefore, the present study was designed to investigate the potentials of the non-edible and disposable part of the oyster mushroom for the adsorption and quantitative removal of atrazine with its three most commonly known degradation products from waste water samples.

## 2 Material and methods

### 2.1 Chemicals and reagents

The analytical standard of *s*-triazine herbicides including atrazine and its most common degraation products such as hydroxyatrazine (ATOH), desethylatrazine (DEA) and desisoopropylatrazine (DIA) were obtained from Dr. Ehrenstorfer (Augsburg, Germany), Table 1. HPLC grade acetonitrile (ACN) was provided by Sigma-Aldrich (France), and was utilized for standard solution preparation of the pesticides and as a mobile phase for chromatographic separation. Methanol was obtained from Carlo Erba (Italy), and used as a mobile phase in the HPLC system. Hydrochloric acid (HCl), received from Sigm-Aldrich (St. Louis, Mo, USA), was used for dissolution of hydroxyl-2-atrazine during stock standard solution preparation and for the pH adjustment of the sample solutions. All the reagents utilized were of analytical grade. Ultrapure water was produced using An 8000 Aquatron water still double distiller (Bibby Scientific, staffordshire, UK) and deionizer (EASYPure LF, Dubuque), and was filtered using cellulose acetate filter paper (0.45 μm, Micro Science) under vacuum.

**Table 1:** General structure and some properties of atrazine and its major degradation products.


**Compound**	**X**	**R**	**R**_**1**_	**Log K**_**ow**_^a^	**pKa**
Atrazine (ATRZN)	-Cl	-NH-CH(CH_3_)_2_	-NH–CH_2_–CH_3_	2.5	1.68
Deethylatrazine (DEA)	-Cl	-NH-CH(CH_3_)_2_	-NH_2_	1.52	1.30–1.65
Deisopropylatrazine (DIA)	-Cl	-NH_2_	-NH–CH_2_–CH_3_	1.15	1.30–1.58
Hydroxyatrazine (ATOH)	-OH	-NH-CH(CH_3_)_2_	-NH–CH_2_–CH_3_	1.4	5.15

a log K_ow_: *n*-octanol–water partition coefficients, defined as the ratio of the equilibrium concentrations of a dissolved substance in two immiscible solvents.

### 2.2 Preparation of pesticide sample solutions

Stock solution containing the mixture of atrazine, DIA, DEA and ATOH was prepared by mixing measured amounts of the individual stadard of pesticide with acetonitrile. However, for dissolution of the hydroxyl-2-atrazine, a known quantity of ATOH was dissolved in 1.0 M HCl (1 mL), and subsequently mixed with the solution of other standards, in acetonitrile [39]. The resulting content was then diluted by acetonitrile to the final volume and stored at 4 °C until its use for adsorption experiements. A 20 mg L^−1^ mixture of working standard solution containing all the analytes were made by diluting the stock solution. A concentrations of standard solution were prepared in a series of 0.125, 0.25, 0.5, 1 and 2 mg L^−1^ to construct a calibration curve.

### 2.3 Instrumentation

Concentrations of the non-adsorbed analytes remained in the supernatant solution were determined utilizing HPLC. The HPLC instrument (Agilent 1200 series) was equiped with Quaternary pump, Vacuum Degasser, Autosampler and Diode Array detector (DAD), all of them are Agilent 1200 Series, were provided by Agilent technologies (Hewlett-Packard-Strasse Waldbronn, Germany). Analytical column, C18 (Techsphere 5 ODS, 25 cm × 4.6 mm ID; HPLC Technology, Macclesfield, Cheshire, UK) was used for chromatographic separation of the target analytes. Anaysis of the resulting data was performed using a B.02.0x revision, Agilent Chem Station software. A various functional groups on the adsorbent was identified by using Spectrum 65 FT-IR spectrometer (PerkinElmer, USA).

### 2.4 Chromatographic conditions

The major chromatographic conditions which can affect separation of different analytes in the column were separately studied and optimized. These chromatographic conditions include selection of wavelength, injection volume, composition of mobile phase, flow rate, and Column temperature. The mobile phase's composition was ACN, methanol and water (22:33:45, v/v/v), adjusted in isocratic mode at a flow rate of 0.5 mL min^−1^. The temperature of the column was kept at 35 °C. The wavelength of detector was set at 230 nm with a bandwidth of 4 and reference wavelength of 360 nm with bandwidth of 100. A 5 μL sample was then injected to the HPLC and allowed to elute for 20 min with 2 min post-time run. Peak area was used as instrument response. A good baseline resolution was achieved for each target analytes under the optimized chromatographic conditions.

### 2.5 Mushroom sample collection and preparation

The samples of Oyster mushroom was obtained from a local farmer, around Addis Ababa city; the capital of Ethiopia, using a pre-cleaned polyethylene bags. The non-edible and disposable part, which is called stalk, was carefully separated from the eidible part using a clean plastic knife. The samples were air dried first, for three days and then dried by oven at 100 °C for 24 h. The resulting mushroom was finally ground using electric mill and kept in a polyethylene bag until used for the experiment.

### 2.6 Bach adsorption study

Sorption experiments were took place at room temperature in batch-mode by agitating 25 mL of the mixture of pesticide standards [40] whose pH were modified utilizing 0.1 M HCl and NaOH in a given volume of flasks, containing 0.06–0.4 g adsorbent masses, on automatic shaker at a shaking speed of 150 rpm. The content was shaken at least for 1 h until the equilibrium was reached and then filtered using whatman No. 42 filter paper, pre-saturated with distilled water [41]. In this manner, the impact of the main experimental conditions including adsorbent dose, pH, initial concentration, contact time and agitation speed on adsorption of atrazine and its major degradation products on mushroom biosorbent were studied. Finally, the pesticide concentrations were calculated from the measured peak area of the chromatographic signals, obtained from diode array detector.

### 2.7 Equilibrium isotherm and kinetic studies

Langmuir and Freundlich isotherm models were employed to understand sorption equilibrium between the analytes (Atrazine, DEA, DIA and ATOH) and the mushroom adsorbent. Similarly, the pseudo-first and -second order models were utilized to study kinetic models [40].

## 3 Results and discussion

### 3.1 Optimization of the chromatographic conditions

**Fig. 1:**
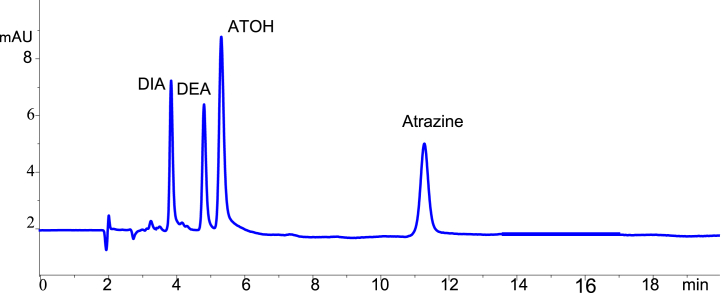
Chromatogram of atrazine, DIA, DEA and ATOH at optimum values of the different parameters. Experimental conditions: mobile phase composition of 33:45:22(*v/v/v)* for methanol/water/acetonitrile at a flow rate of 0.5 mL min^−1^ and detection wavelength of 230 nm.

The composition of mobile phase used throughout this study was acetonitrile, methanol and water (22:33:45 v/v/v) at a 0.5 mL min^−1^ flow rate. The temperature of the column was kept at 35 °C. The wavelength of the detector was set at 230 nm with a bandwidth of 4 and reference wavelength of 360 nm with bandwidth of 100. A 5 μL sample was then injected to the HPLC and allowed to elute for 20 min with 2 min post-time run. Finally, Peak area was used as instrument response. A good baseline resolution was observed for all the target analytes under the optimized chromatographic conditions (Fig. 1).

### 3.2 Characterization of the adsorbent (FTIR analysis)

**Fig. 2:**
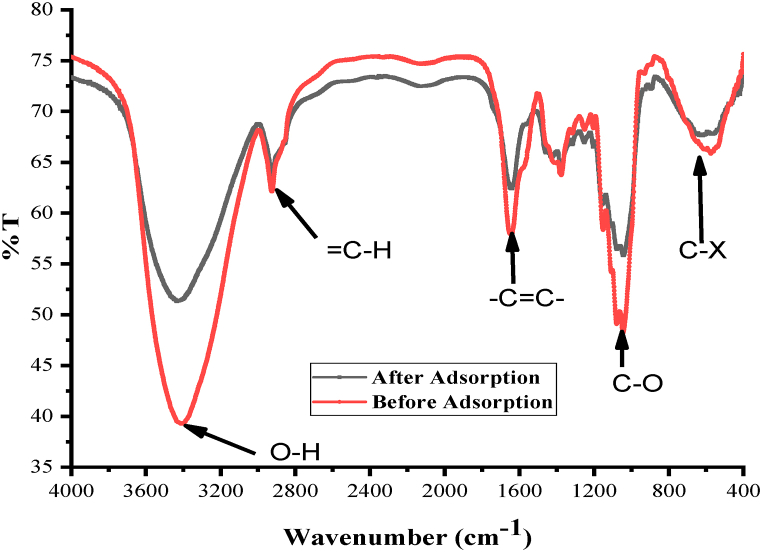
FTIR spectra of oyster mushroom before and after pesticide loading.

The adsorbent's chemical structure is very importan for the better understanding of the sorption mechanism, which is commonly analysed using the FTIR technique. It is very important analytical tool in the identification of functional groups found on the adsorbent surface that are responsible for the overall sorption process [11]. The absorption takes place in the region of IR was attributed to the vibrational and rotational movements of molecular groups and chemical bonds of the molecules [42]. In this study, pesticide loaded and unloaded biosorbents were separately mixed with spectroscopic grade KBr and formed a pellets with the aid of about 1 MPa pressure. The formed pellets had 1 mm thickness and 10 mm diameter. The biosorbent were then scanned in 4000–400 cm^−1^ spectral region [43]. The complex nature of the biosorbent can be observed from the absorption peaks indicated in Fig. 2. The differences in absorption intensities of the different functional groups in the spectra before and after pesticide loading clearly shows that the functional groups were participated in adsorption process (Table 2).

**Table 2:** The FT-IR spectral characteristics of the oyster mushroom before and after pesticide loading.

Before loading		After loading		△** %T	△*υ*	bond	Possible functional groups
*υ*^a^	%T	*υ*	%T				
3412	39	3435	51	12	23	O–H	Phenol, alch.
3150	60	3145	59	−5	−1	= C–H	Alkene
2925	62	2924	64	2	−1	C–H	Aromatics
1643	58	1640	62	4	−3	-C=C-	Alkene
1375	64	1375	65	1	0	C–C (ring)	Aromatics
1044	48	1043	56	8	−1	C–O	Alcohol
574	66	619	68	2	45	C-X	Alkyl halide

a *υ* = Frequency, **△= (After loading – before loading).

### 3.3 Impact of experimental conditions on analyte adsorption

#### 3.3.1 Effect of the pH

**Fig. 3:**
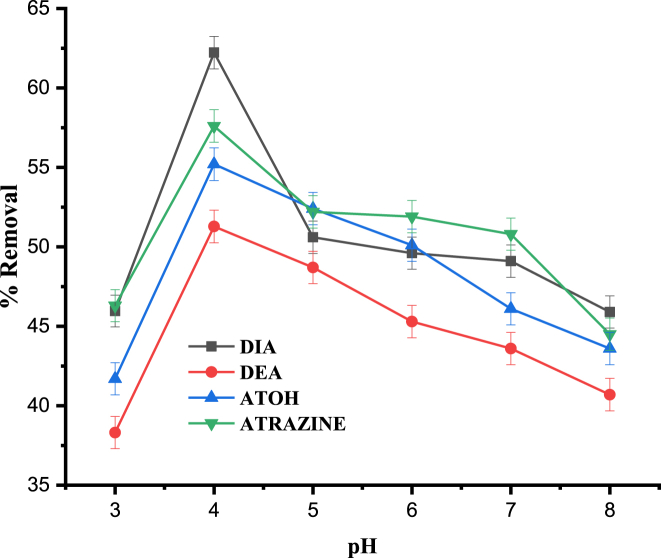
Effect of pH on the percentage removal of DIA, DEA, ATOH and Atrazine. Experimental conditons: contact time, 60 min; agitation speed, 150 rpm; adsorbent dose, 0.2 g; initial concentration, 1 mg L^−1^.

The pH has significant effect on uptake of the analyte molecules primarily because of its influence on the biosorbent's surface characteristics and dissociation/ionization of the biosorbnet molecules [42]. The ionization of sorbent's active functional sites is determined by the pH of the aqueous phase during the adsorption process. This means that at lower pH of extraction solution, surface of the sorbent is predominantly charged positively, whereas at strongly basic pH, the surface is negatively charged, caused by ionization of the functional groups including the hydroxyl, carbonyl, etc groups [36]. In this study, pH was varied from 3 to 8, keeping all the experimental conditions constant, as has also been indicated in Fig. 3. Thus, pH was corrected by making use of 0.1 M HCl and NaOH. Based on these conditions, the maximum removal for atrazine and its degradation products were achieved at pH 4.

#### 3.3.2 Effect of agitation speed

**Fig. 4:**
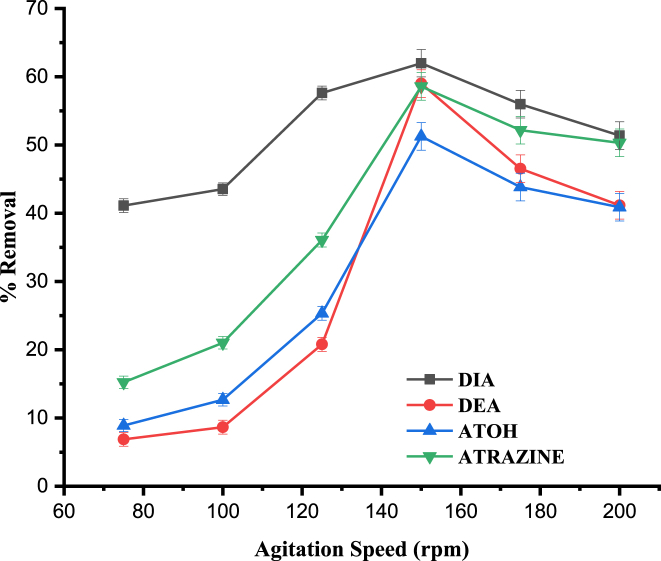
Effect of agitation speed on the percentage removal of DIA, DEA, ATOH and Atrazine. Experimental conditions: pH, 4; contact time, 60 min; adsorbent dose, 0.2 g; initial concentration, 1 mg L^−1^.

Similar to other influencing factors, the speed of agitation also has a surmount effect on the sorption processes, which may be described by the analyte molecules distribution in the bulk solution and formation of the external boundary film [44]. Optimization of the shaking speed in the present study was carried out at 75, 100, 125, 150, 175 and 200 rpm. The percent removal was found to increase with the agitation speed for all the analytes and reached the maximum at 150 rpm, Fig. 4. This may be associated to the phenomena that increasing the speed of agitation decreases the resistance of film to mass transfer surrounding the adsorbent particles and increases mobility of the analyte. Consequently, the adsorbate molecules may be forced towards the surface of the sorbent [37]. On the other hand, increasing the speed beyond 150 rpm, the percent removal started declining which could be associated to the greater tendency of the adsorbed species to undergo desorption at vigorously increased adsorption speed. Thus, speed of 150 rpm was chosen as the optimum for all the subsequent studies.

#### 3.3.3 Effect of contact time

**Fig. 5:**
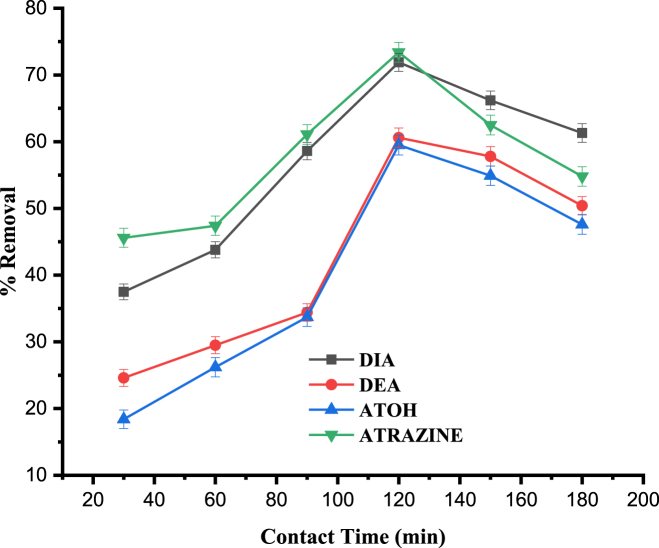
Effect of contact time on the percentage removal of DIA, DEA, ATOH and Atrazine. Experimental conditons: pH, 4; agitation speed, 150 rpm; adsorbent dose, 0.2 g; initial concentration, 1 mg L^−1^.

Contact time is also one of the crucial parameters influencing the batch adsorption process [45]. In the present study, contact time was varied as follows; 30, 60, 90, 120, 150 and 180 min, with all the other experimental parameters kept constant. This was performed to establish the equilibrium time for maximum removal of atrazine and its major degradation products by oyster mushroom. Percent removal of all the analytes increased with time as a result of the available binding sites on the biomass and then reached equilibrium at 120 min, and then remained nearly constant beyond 120 min (Fig. 5). Thus, for all the subsequent experiments, 120 min was selected to ascertain the equilibrium of the target analytes on the adsorbent surface.

#### 3.3.4 Effect of adsorbent dose

**Fig. 6:**
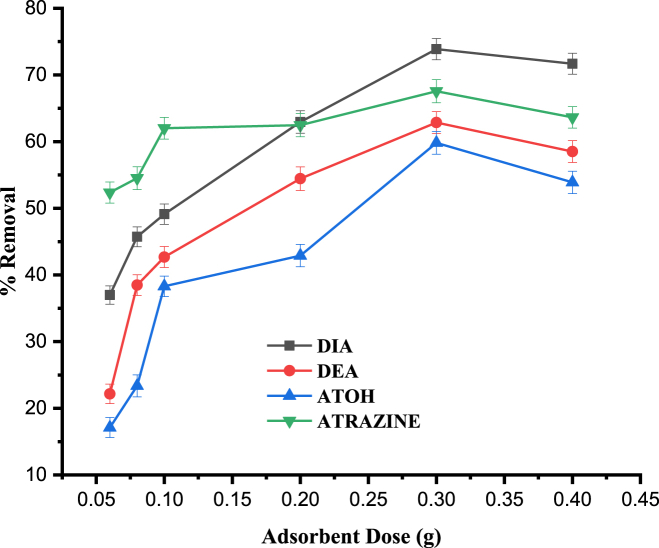
Effect of adsorbent dose on the percentage removal of DIA, DEA, ATOH and Atrazine. Experimental conditons: pH, 4; contact time, 120 min; agitation speed, 150 rpm; initial concentration, 1 mg L^−1^.

The influence of the amount of mushroom sorbent on removal efficiency of the atrazine and its degradation products was investigated using different masses, i.e., 0.06, 0.08, 0.1, 0.2, 0.3 and 0.4 g. These sample doses were equilibrated for 120 min at the pesticide concentration of 1 mg L^−1^, keeping all remaining parameters at their respective optimized values. As can be observed from Fig. 6, increasing the mushroom doses increased the percent removal of all the target analytes. This could be becasue of the increased adsorbent dose, which could result with incrased surface area availability of the adsorbent for facilitating better exposure of the active sites. Moreover, for a constant amount of adsorbate, increasing dose of adsorbent provides enhanced surface area [46]. Similar findings have also been reported by other workers on different types of adsorbate–adsorbent systems [47,48]. However, in the current study, the change in adsoprption of the adsorbent is insignificant and gradually lowered in some instances above the adsorbent dose of 0.3 g. Therefore, the optimum amount of adsorbent used was 0.3 g.

#### 3.3.5 Effect of initial analyte concentration

**Fig. 7:**
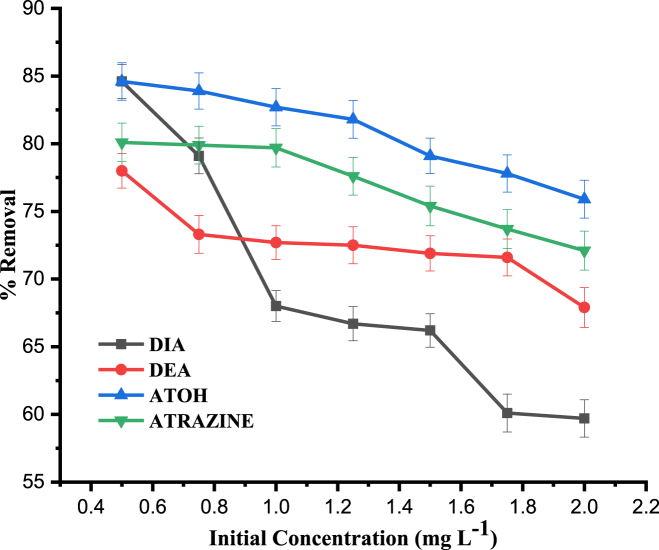
Effect of initial concentration on the percentage removal of DIA, DEA, ATOH and Atrazine. Experimental condditons: pH, 4; contact time, 120 min; agitation speed, 150 rpm; adsorbent dose, 0.3 g.

The influence of the initial analyte concentration on the removal efficiency of atrazine and its degradation products by oyster mushrooms was investigated by varying their initial concentrations as follows; 0.05, 0.075, 0.1, 1.25, 1.50, 1.75 and 2.00 mg L^−1^, at pH 4, contact time of 120 min, shaking speed of 150 rpm and adsorbent dose of 0.3 g. According to the results presented in this study, Fig. 7, the removal of target analyte under study was found to be dependent on the concentration of the respective compounds. It is clear that, the abundance of free binding sites are responsible for the initial high adsorption rate. Moreover, at low concentration, the ratio of available surface to the initial analyte concentration is larger, which results in higher removal of the compounds. However, with increased concentrations of the analytes, this ratio has been declined, and as a result, the analytes removal percentage has been lowered. On the other hand, at higher analytes concentrations, some of the species could be remain in the solution and thus left unadsorbed due to saturation of the binding sites [46].

### 3.4 Adsorption isotherms

The extent of removal of the analytes considered in this study are commonly evaluated using the equation dervied from the sorption equlibria [49]. The analytical results obtained from the equilibrium relationship provide sufficient information on the physico-chemical behaviour of the sorption process. In this context, the amount of adsorbent utilized and that of the analyte in question, at equilibrium, can be related and usually be expressed interms of the sorption isotherm models. The frequently used models, i.e., the Langmuir and Freundlich models, were utilized using mathematical equations for evaluating the precision of the results determined.

#### 3.4.1 Langmuir adsorption isotherm model

It was known that the monolayer coverage of the sorbent surfaces was assumed using Langmuir isotherm model. In addition, interaction of the sorbent in the plane of the adsorbent surface should not be taken in to account [50]. The following mathematical relation gives the linear term of the Langmuir model [51,52].

(1) $$\frac{1}{q_{e}}=\frac{1}{Q_{o}}+\frac{1}{bQ_{o}C_{e}}$$

where, q_e_ is the quantity of the adsorbed analytes (mg g^−1^), C_e_ the concentration of the analyte compounds (mg L^−1^) at equilibrium and Q_o_ and b are the Langmuir constants related to the maximum adsorption capacity (mg g^−1^) and energy of adsorption (mg L^−1^), respectively. The intercept and slope of the plot 1/q_e_ versus 1/C_e_ gives the corresponding values of Q_o_ and b, respectively, as shown in equation (1). In the current study, the corresponding values for Q_o_ and b were found to be (0.994, 0.315), (1.113, 0.132), (0.991, 0.101) and (1.016, 0.252) for DIA, DEA, ATOH and atrazine, respectively, Table 3. In a similar study, heat-treated diatomaceous earth was investigated for its removal efficiency of simazine and atrazine, where removal capacity of the sorbent was found to be 1.3 mg g^−1^ and 0.8 mg g^−1^ for simazine and atrazine, respectively [37]. The most important characteristic of the Langmuir isotherm can be discribed by means of a dimensionless quantity defined as equilibrium parameter or separation factor, R_L_, Table 3. It determines the shape of the isotherm and its values varied in the range from 0 to 1; confirming favorable uptake of the target analytes by the biosorbent. R_L_ can be obtained from equation (2), given below [49].

(2) $$R_{L}=\frac{1}{\left(1+bC_{o}\right)}$$

where, C_o_ is the initial pesticide concentration (mg L^−1^) and b is obtained from the Langmuir plot.

**Table 3:** Langmuir and Freundlich isotherm constants for the adsorption of atrazine and its degradation products on mushroom.

Compound	Langmuir				Freundlich		
	Q_o_	b	R^2^	R_L_	K_F_	1/n	R^2^
DIA	0.994	0.314691	0.9957	0.760635	0.2441	0.8751	0.9913
DEA	1.113	0.131612	0.9750	0.883695	0.1272	0.8934	0.9628
ATOH	0.991	0.101146	0.9832	0.908145	0.0910	0.9301	0.9753
Atrazine	1.016	0.251662	0.9841	0.798937	0.2059	0.8769	0.9756

#### 3.4.2 Freundlich adsorption isotherm model

The Freundlich adsorption isotherm describes the exponential distribution of the active centers, property of heterogeneous surfaces and infinite surface coverage. The linear form of this isotherm is described using the relation given here [53].

(3) Log q_e_ = log K_F_ + (1/n) log C_e_

where, K_F_ (mg g^−1^) and n are the Freundlich constants of the system which describes the sorption capacity, and represents the strength of the adsorptive bond; 'n' is the characteristic constant representing the heterogeneous factor for the bond distribution and adsorption intensity; qe and C_e_ stand for their usual significances, defined earlier. The numerical values of K_F_ and 1/n can be obtained from the straight line obtained by plotting log q_e_ versus log C_e_; with the slope representing 1/n and the intercept log K_F_ (equatioan 3), and the results obtained are given in Table 4. The value of 1/n < 1 indicates that adsorption capacity could slightly be reduced at lower concentration. In this study, close evaluation of the coefficients of variation (R^2^) indicate that the equilibrium data fit better to the Langumuir isotherm model for all the target analytes indicating that monolayer homogeneous surface adsorption expected to dominates.

**Table 4:** Pseudo-first and pseudo-second order adsorption rate constants with the calculated and experimental q_e_ values for adsorption of atrazine and its degradation products.

Compound	q_e_ (mg g^−1^)^a^	Pseudo –First order			Pseudo -Second order			
		q_e_ (mg g^−1^)	K_1_	r^2^	q_e_ (mg g^−1^)	K_2_	h	r^2^
DIA DEA ATOH Atrazine	0.09 0.06 0.07 0.09	0.06 0.04 0.06 0.05	0.0092 0.0078 0.0078 0.0136	0.9572 0.9484 0.9834 0.9954	0.09 0.05 0.07 0.09	0.3827 0.8573 0.2283 0.6049	0.0031 0.0025 0.0011 0.0049	0.9703 0.9989 0.9967 0.9998

a *Experimentally obtained*.

### 3.5 Adsorption kinetics studies

Adsorption kinetics describes the adsorption rate of an adrsorbate on a given adsorbent and is a determining parameter in evaluating adsorption efficiency [52]. Hence, identifying the slowest or the rate determining step is crucial in a given adsorption process. In the current study, two kinetics models, discussed below were applied.

#### 3.5.1 The pseudo-first order model

The pseudo-first order rate equation is expressed using the following relation given in equation (4) [54]:

(4) $$\frac{dq}{dt}=k_{1}\left(q_{e}-q_{t}\right)$$

where, q_t_ and q_e_ are the quantity of each pesticide adsorbed (mg g^−1^) at any time t and at equlibrium, respectively, and k_1_ is the pseudo-first order rate constant (min^−1^). By applying the initial condition, q_t_ = 0 at t = 0, the integrated rate law is;

(5) $$\log\left(q_{e}-q_{t}\right)=\log\;q_{e}-\frac{k_{1}}{2.303}t$$

In the aforementioned relation, the rate of adsorption is considered to be equal to the difference between the adsorption capacity (q_e_) at equilibrium and the capacity at any time t. The values of the coefficient of determination, r^2^, and rate constant, k_1_, could be determined from the plot of straight line of log (q_e_ – q_t_) versus t. In the present study, the k_1_ (min^−1^) and q_e_ (mg g^−1^) values calculaed using the plot described in equation (5) are (0.0092, 0.06), (0.0078, 0.04), (0.0078, 0.06) and (0.01336, 0.05), for DIA, DEA, ATOH and Atrazine, respectively. Similarly, the corresponding values of r^2^, as indicated in Table 4, are 0.9572, 0.9484, 0.9834 and 0.9954 for DIA, DEA, ATOH and Atrazine, respectively.

#### 3.5.2 The pseudo-second order model

The pseudo-second order rate equation is expressed using the following relation [53]:

(6) $$\frac{t}{q_{t}}=\frac{1}{h}+\frac{1}{q_{e}}t$$

where, h = kq_e_^2^ and k (g mg^−1^ min^−1^) is the rate constant for the pseudo-second order adsorption. The values of q_e_ and h are determined from the slope and intercept of the plot of t/qt versus t (equation (6)) and were found to be (0.09, 0.0031), (0.05, 0.00254), (0.07, 0.0011) and (0.09, 0.0049), for DIA, DEA, ATOH and atrazine, respectively, Table 4. The calculated values for the regression coefficient, r^2^, were found to be 0.09703, 0.9989, 0.9967 and 0.9998 for DIA DEA, ATOH and Atrazine, respectively, signifying that the adsorption process follow the pseudo-second order model. This assumption makes use of the fact that chemisorption is the rate determining step [50].

### 3.6 Mechanism for adsorption

The adsorptive removal of a certain pollutant is mostly determined by the properties of the adsorbate and adsorbent, as well as the sorption circumstances. The existence of different types of surface functional groups responsible for the interaction and adsorptive removal of atrazine and its degradation products was identified by FT-IR characterization of the oyster mushroom (Table 4). A proposed mechanism for the adsorption process could be the interaction between amine groups in analyte molecules which are positively charged and the hydroxyl groups that are negatively charged at the adsorbent surface. Furthermore, the occurrence of H-bonds between the amines in analyte molecules and oxygenated groups on the adsorbent surface, including alcohols, may be responsible for adsorption. Similar investigations in the literature have revealed the aforementioned interaction processes for atrazine and its metabolites adsorptive removal utilizing plant-based adsorbents [38,55,56].

## 4 Conclusion

The current study revealed the potential use of the non edible part of oyster mushroom byproduct, as a low-cost biosorbent for the removal of atrazine and its metabolites, i.e., DIA, DEA and ATOH from water samples. The results obtained discribed that the adsorption process of the pesticide compounds by mushroom biosorbent found to depend on pH, contact time, agitation speed, initial herbicide concentration and adsorbent dose. Adsorption capacity of mushroom increased with increase in the concentration of the analytes studied but decreased with increase in pH. Maximum adsorption capacities of 0.994, 1.113, 0.991 and 1.016 mg g^−1^ were found for DIA, DEA ATOH and atrazine, respectively.

The adsorption characteristics was determined by Langmuir and Freudlich isotherm models and it was found that the adsorption process of all the analytes fitted well to Langmuir isotherm indicating a monolayer coverage of the adsorbent surface. The kinetics of the adsorption process was also evaluated using the pseudo-first and -second order models and all the analytes were found to follow the pseudo-second order rate model, showing that the rate limiting step in the adsorption process is chemisorption. Therefore, it may be concluded that the non-edible part of mushroom which is low-cost, easily and abundantly available agricultural product can be utilized for the removal of atrazine and its degradation products from aqueous medium.

## Funding statement

This research did not receive any specific grant from funding agencies in the public, commercial, or not-for-profit sectors.

## Data availability statement

All data used in this scientific works have been included in the body of the manuscript.

## CRediT authorship contribution statement

**Endale Teju:** Writing – original draft, Validation, Conceptualization. **Abi Legesse:** Writing – review & editing. **Negussie Megersa:** Writing – original draft, Visualization, Validation, Supervision, Conceptualization.

## Declaration of competing interest

The authors declare that they have no known competing financial interests or personal relationships that could have appeared to influence the work reported in this paper.
